# A Curious Case of Dengue Fever: A Case Report of Unorthodox Manifestations

**DOI:** 10.1155/2020/1701082

**Published:** 2020-07-25

**Authors:** Raja Shakeel Mushtaque, Syed Masroor Ahmad, Rabia Mushtaque, Shahbano Baloch

**Affiliations:** ^1^Jinnah Post Graduate Medical Center, Karachi, Pakistan; ^2^National Institute of Cardiovascular Diseases, Karachi, Pakistan

## Abstract

Dengue is the major cause of arthropod-borne viral disease in the world. It presents with high fever, headache, rash, myalgia, and arthralgia and it is a self-limiting illness. Severe dengue can occur in some cases resulting in dengue hemorrhagic fever (DHF) and dengue shock syndrome (DSS). We present a case of a 32-year-old male patient of high-grade fever, bilateral subconjunctival hemorrhages, swelling on hands and lips, and nasal bleeding. After investigations, he was diagnosed with dengue fever and it was observed that he developed systemic fungal infection secondary to *Candida tropicalis* infection. The patient's bone marrow biopsy showed hemophagocytic activity. He also developed hepatitis E infection while hepatitis A, B, or C serology profile showed no active infection. The bilateral iliopsoas hematoma was also observed on CT scan manifested by decreased power in bilateral lower limbs and pain in the right leg. The patient was treated in the hospital with antibiotics (ceftriaxone 2 g once daily for 14 days) and antifungal (fluconazole 200 mg per oral initially for one day then 100 mg daily for 13 days) medicines, and his condition improved on discharge. There is evidence of variable presentations of dengue fever after the disease burden is increased, and thus, diagnosing with such manifestations can be very challenging.

## 1. Background

Dengue is the foremost cause of arthropod-borne viral disease in the world and due to severe muscle aches. It is transmitted through *Aedes* mosquito and commonly found in tropical and subtropical parts of the world. The incidence of dengue has substantially increased over the past few decades [1]. It was estimated in a study that 3.9 billion people are at risk of infection with dengue viruses in the world and Asia is the most affected part [2]. A seasonal pattern of dengue linked to climate. In Pakistan, the highest dengue cases are reported during July-September due to more rainfall, optimum temperature, and humid environment which are ideal for breeding of *Aedes* mosquitoes [3]. Last year, the outbreak was first reported on 8 July 2019 in Peshawar city. A total of 47,120 confirmed cases of dengue fever, including 75 deaths, were reported during the outbreak period in the entire country [4].

Dengue fever is caused by one of the four distinct serotypes (DENV 1-4) of single-stranded RNA *Flavivirus* genus [5]. Infection caused by one serotype results in lifelong immunity to that serotype, but not to others. Dengue fever (DF) presents with high fever, headache, rash, myalgia, and arthralgia, and case fatality is less than 1%. Severe dengue, dengue hemorrhagic fever (DHF), and dengue shock syndrome (DSS) are accompanied by thrombocytopenia, vascular leakage, and hypotension [5]. DSS is characterized by systemic shock, which can be fatal with case fatality high as 12% to 44% [6].

There are few atypical manifestations of dengue fever growing with rising disease burden, often missed and sometimes difficult to comprehend the case collectively. In this case report, we will discuss atypical manifestations observed in dengue fever patients.

## 2. Case

A 32-year-old male patient, married, with no previous comorbidities presented through the emergency room with complaints of fever for 8 days, bilateral subconjunctival hemorrhages, and swelling of hands and lips for 3 days and 1 episode of nasal bleed one day back. Fever was high grade, continuous, and associated with rigors/chills and generalized body ache. The patient developed bilateral conjunctival hemorrhages that were all of sudden, not associated with any trauma. The patient also had swelling on both hands and lips, which progressed over the 3 days. He also had one episode of nasal bleeding that was all of sudden and 1-2 teaspoons in quantity. He also had a tingling sensation in both lower limbs and difficulty in walking. He denied any history of bleeding from other parts of the body or any vomiting. He denied any previous hospitalization or any drug intake or any substance abuse. Her family came from a middle‐class background, he was sexually active with his wife, and he denied any chronic illness in his family.

## 3. Examination

A middle-aged male patient, ill-looking but oriented with time, place, and person. His vitals at the time of examination were blood pressure of 110/80 mmHg and pulse of 82/min regular, and the respiratory rate was 20/min. He was febrile (101°F), anemic, and jaundiced, while his hands and legs were mildly edematous, and lips were mildly swollen up and bilateral subconjunctival hemorrhage was noted. His abdomen was soft, slightly tender over the right hypochondrium, and mildly distended, and the liver was enlarged 3 cm below the right costal margin, firm, and nonnodular. On auscultation of the chest, there was normal vesicular breath sounds bilaterally except decreased breath sounds over the right base. The examination of the cardiovascular system was unremarkable.

On neurological examination, his GCS was 15/15, there were no signs of meningeal irritation, pupils were 3 mm bilaterally equally reactive to light and accommodation, and cranial nerves were intact. On motor examination, muscle bulk and tone were normal. Power in the lower limb was reduced, but normal in the upper limbs. Medical Research Council (MRC) grade in the right lower extremity was hip flexion 3/5 and extension 3/5, hip abduction 4/5 and adduction 4/5, and leg flexion 3/5 and extension 3/5 and dorsiflexion and plantar-flexion 5/5 strength bilaterally. MRC grade in the left lower extremity was hip flexion 4/5 and extension 4/5, hip abduction 5/5 and adduction 5/5, and leg flexion 4/5 and extension 4/5 and dorsiflexion and plantar-flexion 5/5 strength bilaterally. Additionally, there was a pain in the distribution of dermatome L2, L3, and L4 on the right lower limb. Deep tendon reflexes on the right lower cannot be performed due to pain, although they were normal and 2+ on the left side, and planters were bilaterally downgoing. The sensation was intact throughout, and the cerebellar examination was normal.

## 4. Differential Diagnosis

As this patient presented with high-grade fever, subconjunctival hemorrhage, nasal bleeding, and hepatosplenomegaly, a provisional diagnosis of viral hemorrhagic fever was made. The other differentials included malaria, viral hepatitis, and leptospirosis.

## 5. Investigation

Baseline laboratory investigations are shown in Table 1, and hepatitis virology and autoimmune work are given in Table 2. Serum dengue NS-1 antigen was positive while the MP-ICT was negative. Peripheral smear of CBC showed a leukoerythroblastic picture. Platelet clumps were observed, and anisocytosis, poikilocytosis, polychromasia, macrocytes, nucleated RBC, myelocytes, and metamyelocytes were seen.

Urine analysis was suggestive of urinary tract infection (nitrate 1+, leukocytes: 40–50/HPF, RBC: 20–25/HPF, epithelial cells: ++/HPF, and casts: nil), but no organism grew on culture studies. The blood culture grew *Candida tropicalis*, which was sensitive to fluconazole and voriconazole. His bone marrow biopsy report showed preserved trilineage hematopoiesis along with the hemophagocytic activity. Leptospirosis serology was unremarkable.

## 6. Imaging

Computed tomography (CT) scan of the chest and abdomen revealed bilateral pleural effusions with adjacent patchy consolidations and hepatosplenomegaly along with moderate ascites was noted. Diffuse thickening of bilateral iliopsoas muscles was also noted with areas of internal necrosis and ill-defined heterogeneous enhancement. His ultrasound Doppler of both legs was unremarkable except mild soft-tissue edema.

## 7. Treatment and Follow-Up

The patient was started on intravenous antibiotics (ceftriaxone 2 g once daily for 14 days) and antifungal (fluconazole 200 mg per oral initially for one day then 100 mg daily for 13 days). He was symptomatically managed with intravenous fluids (0.9% normal saline), antipyretics (paracetamol), and antiemetics (inj. gravinate[dimenhydrinate]). He improved with the treatment. He was discharged home after three weeks. At the time of discharge, his fever was subsided, bilateral hand and feet swelling subsided, and subconjunctival hemorrhage resolved, but he still complained of pain during walking and lower limb weakness. On the first clinic follow-up visit, his leg pain and weakness improved.

## 8. Discussion

This patient's complete blood picture showed increased total leukocyte count (TLC): 25.2 (neutrophils 40% and lymphocytes 57%), and later, the blood culture was positive for *Candida tropicalis*. The phenomenon of bacteremia in dengue has been identified and can occur simultaneously with various organisms like *Streptococcus pneumoniae*, *E. coli*, *Salmonella* species, *Shigella sonnei*, *Klebsiella* species, *Enterococcus faecalis*, *Moraxella lacunata*, *Staphylococcus aureus*, *Haemophilus influenzae*, *Candida tropicalis*, *Mycobacterium tuberculosis*, *Mycoplasma*, or *Herpesviruses* [7]. *Candida tropicalis* normally inhabit the skin and intestinal tract. There is evidence of intestinal mucosal injury in patients with dengue infection. Therefore, the vulnerability of intestinal mucosa due to dengue virus infection may lead to the transfer of organisms into the bloodstream [8].

The peripheral smear of complete blood count of this patient showed the leukoerythroblastic picture, and bone marrow biopsy showed preserved trilineage hematopoiesis along with the hemophagocytic activity. Hemophagocytic lymphohistiocytosis (HLH) (also known as “hemophagocytic syndrome”) is a hyperinflammatory condition characterized by sustained activation of the mononuclear phagocytic system that may result in a severe hyperinflammatory response. Epstein–Barr virus (EBV-HLH) is a recognized cause of acquired HLH [9], but it has also been reported as a complication of dengue. In a retrospective study carried out in Malaysia, adult patients with severe dengue showed HLH in twenty-one patients of 180 (12%) [10]. In another study, a total of 33 HLH patients were identified, of which 22 (67%) were associated with dengue and 1 died [11]. HLH was not found to be associated with a particular type of dengue virus. These patients had a longer duration of fever and were more likely to have anemia, hepatomegaly, and elevated liver transaminases than controls.

In this case report, there was no active infection of hepatitis A or B or C infection, but the patient had acute hepatitis E infection as shown by hepatitis virology and autoimmune workup in Table 2. The presenting signs and symptoms were overlapping between viral hepatitis and dengue fever, and thus, it could be hard to challenging diagnosis in an endemic area. In one study, it was found that women were infected with dengue virus and hepatitis E virus simultaneously [12]. Another study was conducted in India found that a young man was infected with dengue, HEV, and *Leptospira* at the same time [13]. In epidemic regions, a physician should be vigilant for identifying such coinfections.

Our patient had decreased hip flexion and extension, hip abduction and adduction, and leg flexion and extension on the right leg, while on the left side, hip flexion and extension and leg flexion and extension were affected. There was also the pain in the distribution of dermatome L2, L3, and L4 on the right lower limb. On CT scan, there was diffuse thickening of the bilateral psoas muscle and iliacus muscles noted with areas of internal necrosis and ill-defined heterogeneous enhancement noted, pointing towards bilateral iliopsoas hematoma. Iliopsoas hematomas have also been associated with compressive femoral neuropathy due to the long course of the femoral nerve. Iliopsoas hematoma is usually caused by trauma in patients on anticoagulation/antiplatelet therapy or in those with hemophilia [14]. In 1939, Tallroth first ever explained the occurrence of spontaneous hemorrhage in the iliopsoas muscle followed by an injury to the femoral nerve in a hemophilia patient. Muscle hematomas are a rare complication of dengue fever. Only a few cases have been reported in the literature of spontaneous muscle hematomas in DHF reported by Ammer et al. [15], Ganeshwaran et al. [16], Ganu and Mehta [17], Koshy et al. [18], and Kumar et al. [19]. The hematoma could have resulted from thrombocytopenia and deranged international normalization ratio (INR), thus resulting in hemorrhage.

## 9. Learning Points

1. Bacteremia or systemic fungal infections can occur in dengue fever. Thus, it should be looked at with high suspicion to avoid morbidity and mortality.
2. Hemophagocytic lymphohistiocytosis (HLH) can occur in dengue fever and can cause high mortality.
3. Coinfection of dengue fever can occur with hepatitis E and cause overlapping symptoms which can cause a challenging situation for a physician to diagnose.
4. Dengue fever can present with bilateral iliopsoas muscle hematoma with femoral nerve palsy. This phenomenon was only reported a few times.

## Figures and Tables

**Table 1:** Baseline laboratory investigations.

Laboratory investigations	Results	Normal value
Hb	7.0	13.5 to 17.5 g/dl
MCV	103.9	80–95 fL
TLC	25.2	4.5–11.0 × 109/L
Neutrophils	40%	55–70%
Lymphocytes	57%	20–40%
Platelets	137 × 10E9/L	150–400 × 109 per liter
Total bilirubin	4.75	0.1–1.0
Direct bilirubin	2.49	0.1–0.4
GGT	244	9 to 85 IU/L
ALT	482	7–56 IU/L
ALP	1397	41 to 133 IU/L
Total albumin	5.98	6 to 8 g/dl
Globulin	3.03	2.0 to 3.5 g/dL
Albumin	3	3.5 to 5.0 g/dl
Prothrombin time	59.4	11.0–12.5 seconds
INR	5.30	0.8–1.1
APTT	52.1	30–40 seconds
LDH	13120 U/L	140 U/L 280 U/L
D-Dimer	0.7 mg/dl	Less than 0.3
Serum B12 level	1471 pg/ml	191–663
ESR	53 mm/hr	0–25
Reticulocyte count	6.8%	0.5% to 2.5%

Hb: hemoglobin; MCV: mean corpuscular volume; TLC: total leukocyte count; GGT: gamma-glutamyltransferase; ALT: alanine transaminase; ALP: alkaline phosphatase; INR: international normalization ratio; APTT: activated partial thromboplastin time; LDH: lactate dehydrogenase; ESR: erythrocyte sedimentation rate.

**Table 2:** Hepatitis virology and autoimmune workup.

Laboratory investigations	Results
Hepatitis A	
IgM	Nonreactive
IgG	Reactive
HBsAg	Nonreactive
Hepatitis B core antibodies	
IgM	Both nonreactive
IgG	
Anti-hepatitis C	Nonreactive
Hepatitis E	
IgM	Both reactive
IgG	
ANA	Negative

IgM: immunoglobulin M; IgG: immunoglobulin G; HBsAg: hepatitis B surface antigen; ANA: antinuclear antibodies.
